# *De novo* Comparative Transcriptome Analysis of Genes Differentially Expressed in the Scion of Homografted and Heterografted Tomato Seedlings

**DOI:** 10.1038/s41598-019-56563-z

**Published:** 2019-12-27

**Authors:** Hui Wang, Peng Zhou, Wenying Zhu, Fu Wang

**Affiliations:** College of Horticulture, Qingdao Agricultural University, Qingdao, 266109 China

**Keywords:** Transcriptomics, Plant molecular biology

## Abstract

Tomato is an important vegetable crop grown worldwide. Grafting is an agricultural technique that is used to improve growth, yield, and resistance to diverse stresses in tomato production. Here, we examined the differences between the scion of heterografted (‘Provence’/‘Haomei’) and homografted (‘Provence’/‘Provence’) tomato seedlings. We observed anatomical changes during the graft-union healing process in heterografted and homografted tomato seedlings and conducted transcriptome analyses of the ‘Provence’ scion from both graft combinations. With the development of calli from both graft partners, the isolation layer became thinner at 16 d after grafting (DAG). Compared with that of homografts, the healing in heterografts was slightly delayed, but the graft union had completely healed at 21 DAG. In total, 858 significantly differentially expressed genes were detected between the transcriptomes of heterografts and homografts at 16 DAG. Functional pathways identified by GO and KEGG enrichment analyses were associated with primary and secondary metabolism, hormone signalling, transcription factor regulation, transport, and responses to stimuli. Many differentially expressed genes were involved in pathways associated with mitogen-activated protein kinase signalling, plant hormone signalling, and oxidative stress. A number of transcription factors were up-regulated in the scion of heterografted seedlings. The results provide a valuable resource for the elucidation of the molecular mechanisms, and candidate genes for functional analyses, of heterograft and homograft systems.

## Introduction

Tomato (*Solanum lycopersicum*), a member of the Solanaceae family, is among the most widely cultivated vegetables and has high economic and nutritional values. In 2016, the planting area of tomato worldwide was 4.8 million ha, with a yield of 177 million t^1^. Given the high market demand for off-season vegetables, crops are continually produced under unsuitable conditions, leading to increased incidences of physiological and pathological disorders that result in crop yield losses^2,3^. Grafting is an important technique used widely for vegetable production to enhance plant vigour through the avoidance of soil pathogens and the increased tolerance to low temperature, high salinity, and drought^4–8^. Grafted tomato plants develop a stronger root system, which can improve the utilization of fertilizers (especially nitrogen fertilizer) and water, thereby improving fruit quality and yield^9–11^. Hence, grafting has not only a major impact on tomato growth and development, but also has positive effects on fruit quality, such as dramatically improved flavour and increased quantities of health-related compounds^7,11^.

The majority of reports on plant grafting in the past decades have focused on grafting’s physiological processes and histological characteristics^12–15^. Several previous studies investigated the graft healing process^16,17^, which involves the following sequence of processes: (1) initial adhesion and formation of the isolation layer between the scion and the rootstock; (2) formation of calli; and (3) reconnection of vascular bridges. During the graft healing process of tomato, Zhao^18^ observed that an isolation layer and a small number of callus cells formed in the graft union by 5 days after grafting (DAG) and that a large number of callus cells and vascular bundle reconnections appeared during the late stage of healing.

Increasingly, related research has focused on the elucidation of potential molecular mechanisms involved in grafting using genetic approaches. Grafting likely involves a complex signalling system that regulates genetic mechanisms associated with graft healing in vegetable crops^19^. Previous studies have reported the detection of the long-distance transport of certain molecules, especially phytohormones, RNAs, peptides, and proteins, after grafting^4,12,20,21^. Through these studies, significant breakthroughs have been achieved in uncovering the underlying mechanisms implicated in the regulation of development and stress responses at the whole-plant level. Nevertheless, the molecular mechanisms involved in the grafting of tomato remain incompletely understood, especially between homo- and heterograft combinations.

The availability of the tomato genome sequence and the development of RNA sequencing (RNA-Seq) technology are ideal tools for the identification and analysis of important genes at the transcriptional level^22^. Advances in sequencing technology have presented opportunities for genomic analyses and the investigation of gene functions in both model and non-model organisms. Among next-generation sequencing technologies, RNA-Seq is the most powerful tool currently available for comparative transcriptome profiling^23,24^. The procedure is highly reproducible, with few systematic discrepancies detected among technical replicates^25^. The transcriptome analysis of grafted plants may reveal specific genes involved in the regulation of grafting-induced physiological responses^19^.

In this study, scions of tomato ‘Provence’ were grafted onto tomato ‘Haomei’ rootstocks, and self-grafted ‘Provence’ was used as the control. Based on the time course of the graft healing process, the scion at 16 DAG was selected for RNA-Seq. The transcriptomic data were analysed using bioinformatics tools to determine the transcriptional network and major metabolic activities involved in rootstock-mediated effects on plant growth, development, and acclimation to environmental stresses. In addition, we validated the transcriptome results using a quantitative real-time PCR (qPCR) analysis. This study provides a foundation for the elucidation of the differentially expressed genes (DEGs) in the scion of hetero- and homografted seedlings.

## Results

### Microscopic observation of graft healing

**Figure 1:**
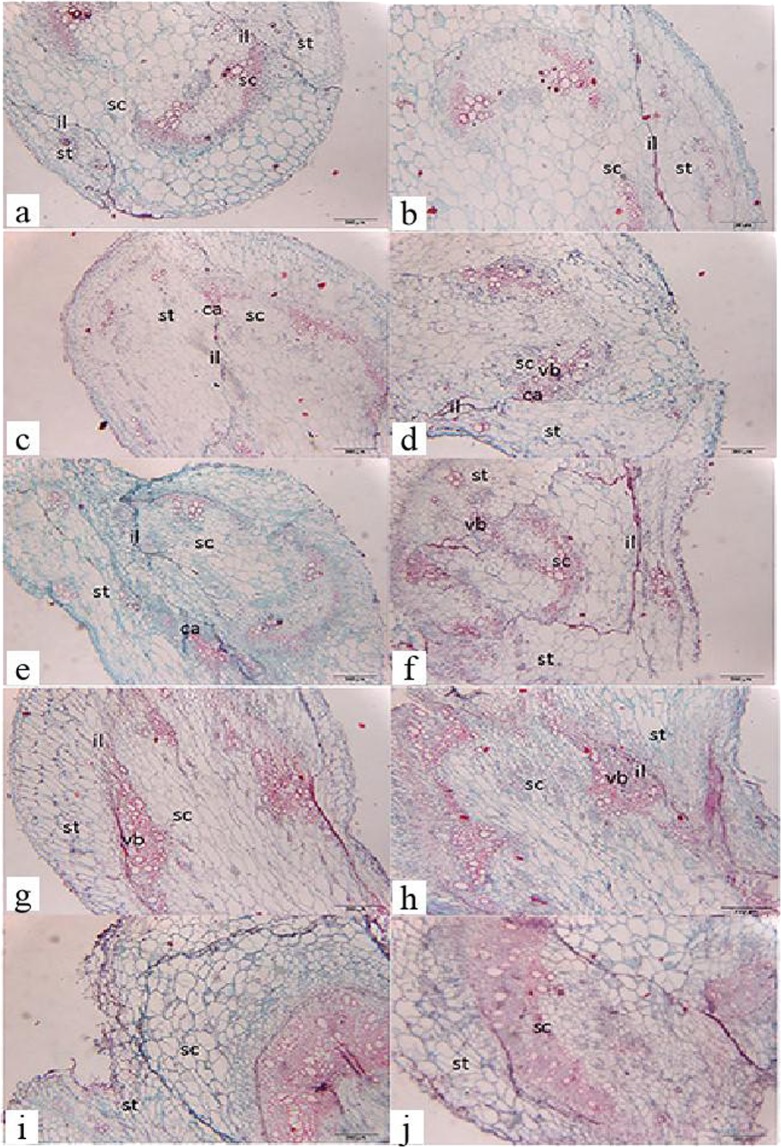
Anatomical progression of graft healing in grafted tomato seedlings. (**a**,**c**,**e**,**g**,**i**) Transverse sections of the graft union of the ‘Provence’/‘Haomei’ (stock/rootstock) combination at 6, 9, 12, 16, and 21 days after grafting (DAG), respectively. (**b**,**d**,**f**,**h**,**j**) Transverse sections of the graft union of the ‘Provence’/‘Provence’ combination at 6, 9, 12, 16, and 21 DAG, respectively. st: stock; sc: scion; il: isolation layer; vb: vascular bridge; ca: callus. Bars = 200 μm.

At 6 DAG, the isolation layer had developed and delimited the adjacent tissues of both partners in the ‘Provence’/‘Haomei’ and ‘Provence’/‘Provence’ grafted seedlings. Additionally a small amount of callus had developed (Fig. 1a,b). At 9~12 DAG, the amount of callus had increased in both graft combinations, but a large number of vascular bridges appeared in the homograft (Fig. 1c–f). At 16 DAG, the isolation layer was thinner and a large number of vascular bridgings were visible in the hetero- and homografted seedlings. We also noted that the vascular bundles were reconnected between the rootstock and the scion at this point, but no differences in the levels of the graft-union healing between hetero- and homograft combinations were observed (Fig. 1g,h). With the proliferation and differentiation of callus cells between hetero- and homografts, the graft boundary was almost absent, and the graft union was completely healed, at 21 DAG (Fig. 1i,j). Compared with the healing of the heterografted seedlings, that of the homograft union was significantly accelerated. Because gene expression levels usually show significant differences prior to microscopic morphological changes, we selected grafted seedlings at 16 DAG for an RNA-Seq analysis combined with the anatomical observations.

### RNA-Seq data analysis

To explore differences in the molecular mechanisms of the scion between hetero- and homograft tomato seedlings, we used Illumina sequencing technology to analyse the transcriptome profiles of the scion ‘Provence’. A total of 83,123,467 raw reads were obtained. Approximately 81,081,965 clean reads with >90% Q30 bases (those with a base quality greater than 30) were selected as high-quality reads for further analysis (Table 1). The high-quality reads were mapped to the reference tomato transcript sequences, resulting in the mapping of approximately 96% of the nucleotides. Mapping revealed that transcripts of 22,740 (96.82%) and 22,230 (96.9%) genes were detected in the scions of hetero- and homografted seedlings, respectively (Table 2).

**Table 1:** RNA sequencing data statistics.

	Provence/Haomei	Provence/Provence
Raw Reads Number	41567013	41556454
Clean Reads Number	40605151	40476814
Clean Reads Rate(%)	97.68	97.39
Low-quality Reads Number	174961	153579
Ns Reads Number	848	827
Adapter Polluted Reads Rate(%)	1.89	2.23
Clean Q30 Bases Rate(%)	93.04	93.63

**Table 2:** Mapping of RNA-Seq high-quality reads.

Sample	Total clean reads	Mapping rate(%)	Total gene number
Provence/Provence	40476814	96.90	22230
Provence/Haomei	40605151	96.82	22740

### Functional annotation and classification of DEGs

**Figure 2:**
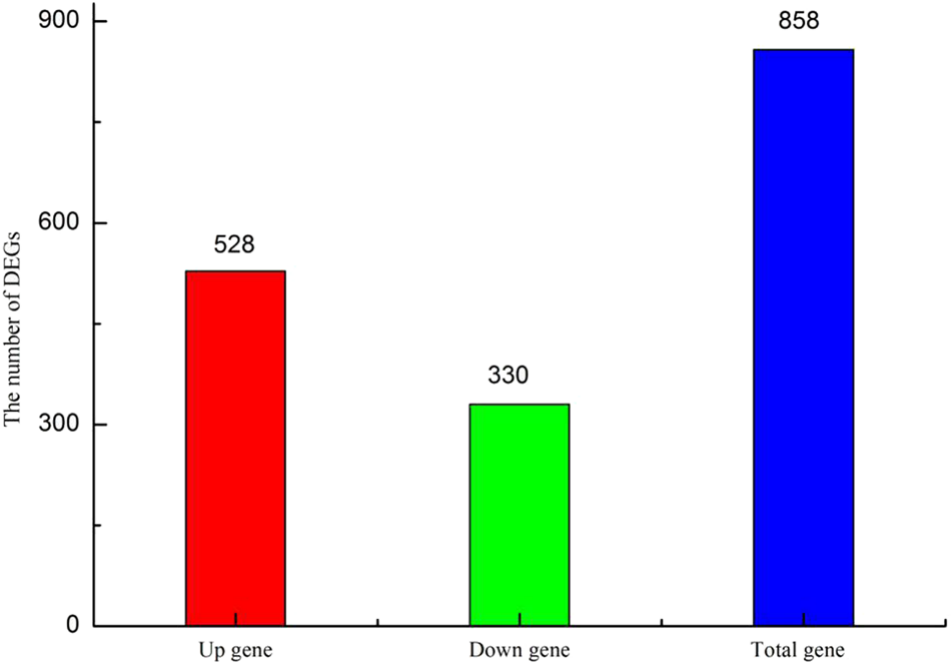
Number of differentially expressed genes (DEGs) detected between hetero- and homografted tomato seedlings.

To identify the DEGs between the control (homografted seedlings) and heterografted seedlings, we employed a general chi-squared test with false discovery rate (FDR) correction and a *P*-value of 0.05 using DEseq6 software to identify twofold up-regulated and twofold down-regulated genes. In total, 858 significantly DEGs were detected between the control and the treatment samples, with 528 up-regulated genes and 330 down-regulated genes being detected in the heterografted samples (Fig. 2).

To explore the possible functions of the DEGs, BLAST searches of the non-redundant protein (NR), nucleotide sequences (NT), Clusters of Orthologous Groups (COG), UniProt, gene ontology (GO), and Kyoto Encyclopedia of Genes and Genomes (KEGG) databases were performed. The annotated genes that showed significant similarities in each database were 838 (96.67%), 858 (100%), 707 (82.4%), 297 (34.61%), 570 (66.43%), and 273 (31.81%), respectively (Table 3). Overall, 858 DEGs were annotated based on information in one or more of the NR, NT, UniProt, GO, KEGG, and COG databases.

**Table 3:** Functional annotation of the differentially expressed genes (DEGs) detected between hetero- and homografted tomato seedlings.

	DEGs number(n)	Percentage(%)
total gene	858	100
Annotated in Nr	838	97.67
Annotated in Nt	858	100
Annotated in Uniprot	707	82.4
Annotated in CoG	297	34.61
Annotated in GO	570	66.43
Annotated in KEGG	273	31.81

### GO enrichment analysis of DEGs

**Figure 3:**
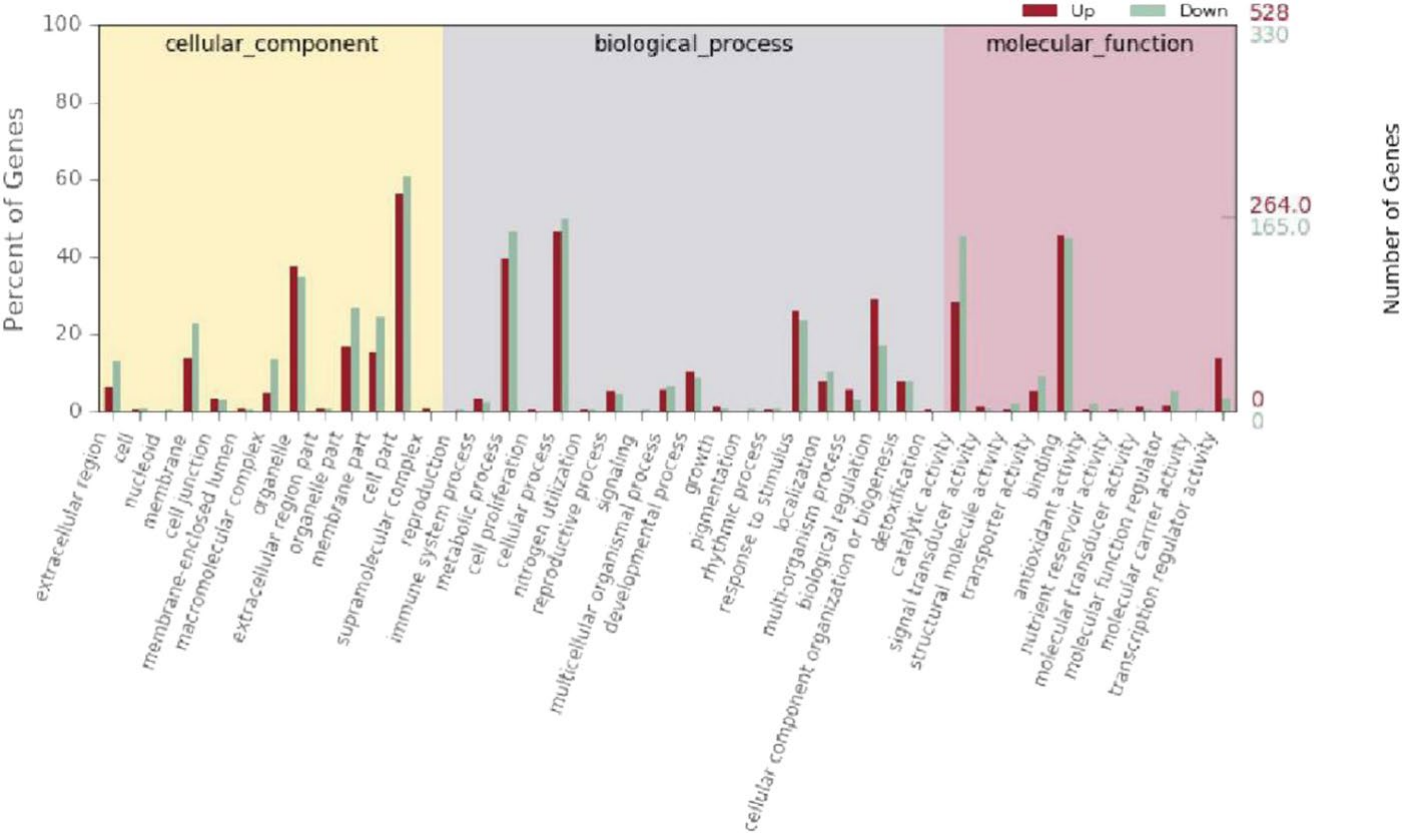
Gene ontology (GO) statistics and enrichment analysis of identified differentially expressed genes (DEGs) detected between hetero- and homografted tomato seedlings.

For the functional classification of the DEGs, a GO enrichment analysis was performed. On the basis of the sequence alignments, 858 DEGs were classified into 43 functional groups belonging to three main categories: cellular components, molecular functions, and biological processes (Fig. 3). The GO functions were significantly enriched in the scion of the heterografted seedlings

In the cellular components category, ‘cell part’, ‘organelle’, and ‘membrane terms’ were significantly enriched. In the biological processes category, ‘metabolic processes’, ‘cellular processes’, ‘response to stimulus’, and ‘biological regulation’ were significantly enriched. In the molecular functions category, ‘catalytic activity’ and ‘protein binding’ were significantly enriched. In addition, a number of DEGs were classified into two functional subclasses involved with transcription regulator activity and transporter activity. Thus, the majority of the identified DEGs were responsible for fundamental processes associated with biological regulation and metabolism (Fig. 3).

### KEGG enrichment analysis of DEGs

**Figure 4:**
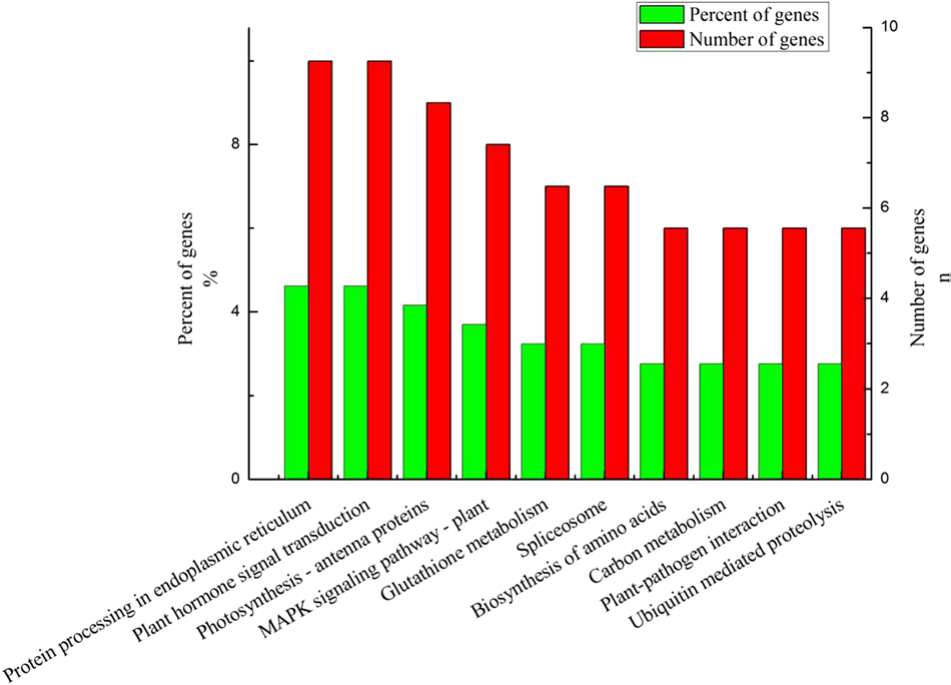
The 10 most significantly enriched KEGG pathways among differentially expressed genes detected between hetero- and homografted tomato seedlings.

A KEGG pathway enrichment analysis was performed to categorise the biological functions of DEGs. A total of 858 DEGs were allocated to 87 KEGG pathways (Supplementary Table S1). Interestingly, the pathways having the highest numbers of DEGs were protein processing in the endoplasmic reticulum and plant hormone signal transduction, followed by photosynthesis-antenna proteins, mitogen-activated protein kinase (MAPK) signalling pathway-plant, glutathione metabolism, biosynthesis of amino acids, and carbon metabolism (Fig. 4).

### Analysis of DEGs between hetero- and homografted seedlings in the MAPK and plant hormnone signal pathways

In the present study, genes associated with MAPK signalling pathways were identified. The significant up-regulation of genes in the MAPK signalling pathways is associated with pathogen infection, plant hormones, and wounding.

In the current transcriptome analysis, *WRKY33/PP2C* genes (*Solyc09g014990* and *Solyc07g040990*) associated with pathogen infection, genes associated with plant hormones, such as ethylene receptors (*Solyc06g053710* and *Solyc09g075440*), an ethylene-responsive transcription factor 1 (*Solyc09g089930*), serine/threonine-protein kinase SRK2 (*Solyc08g077780*), and an abscisic acid (ABA) receptor in the PYR/PYL family (*Solyc08g076960*), were significantly up-regulated (Table 4). In addition, a gene for respiratory burst oxidase (*Solyc08g081690*) expressed in wounds was up-regulated.

**Table 4:** Data of selected differentially expressed genes (DEGs) associated with the MAPK signalling pathway detected between hetero- and homografted tomato seedlings.

Genes	Log_2_^FoldChange^	Up/Down	KEGG:KO	KEGG:Annotation
**pathogen infection**				
*Solyc09g014990*.*2*	3.12	up	K13424	WRKY33; WRKY transcription factor 33
*Solyc07g040990*.*2*	1.39	up	K14497	PP2C; protein phosphatase 2 C
**Ethylene**				
*Solyc06g053710*.*2*	1.23	up	K14509	ETR, ERS; ethylene receptor
*Solyc09g075440*.*2*	1.18	up	K14509	ETR, ERS; ethylene receptor
**ABA**				
*Solyc09g089930*.*1*	3.24	up	K14516	ERF1; ethylene-responsive transcription factor 1
*Solyc08g077780*.*2*	1.70	up	K14498	SNRK2; serine/threonine-protein kinase SRK2
*Solyc08g076960*.*1*	1.36	up	K14496	PYL; abscisic acid receptor PYR/PYL family
**wongding**				
*Solyc08g081690*.*2*	1.33	up	K13447	RBOH; respiratory burst oxidase

The majority of genes associated with the regulation of diverse hormones was differentially expressed between hetero- and homografted seedlings (Table 5). The transcriptome analysis showed that the expression of genes associated with the auxin, gibberellin, ABA, ethylene, and jasmonic acid signalling pathways changed significantly. We speculated that these hormone signalling pathways might be involved in differences in graft healing between hetero- and homografted tomato seedlings. The transcript levels of most auxin transporter-encoding genes changed significantly in the scion of the heterograft combination. For example, the gene *Solyc11g013310*.*1*, a member of the auxin influx carrier family, was down-regulated, whereas the gene *Solyc07g014620*.*1* (a SAUR family protein) was up-regulated. The most prominent role of gibberellin is to accelerate cell elongation and promote cell division. The *PIF3* gene (*Solyc01g102300*.*2*) associated with gibberellin signalling was up-regulated in the scion of the heterograft combination. In addition, we identified six up-regulated genes and one down-regulated gene involved in the ABA, ethylene, and jasmonic acid signalling pathways in the scion of the heterograft combination.

**Table 5:** Data of selected differentially expressed genes (DEGs) associated with plant hormone signal transduction pathways detected between the hetero- and homografted tomato seedlings.

Genes	Log_2_^FoldChange^	Up/Down	KEGG:KO		KEGG:Annotation
**auxin**					
*Solyc07g014620*.*1*	1.23	up	K14488	SAUR; SAUR family protein	
*Solyc11g013310*.*1*	−1.01	down	K13946	AUX1, LAX; auxin influx carrier (AUX1 LAX family)	
**Gibberellin**					
*Solyc01g102300*.*2*	2.00	up	K12126	PIF3; phytochrome-interacting factor 3	
**Abscisic acid**					
*Solyc08g077780*.*2*	1.70	up	K14498	SNRK2; serine/threonine-protein kinase SRK2	
*Solyc07g040990*.*2*	1.39	up	K14497	PP2C; protein phosphatase 2 C	
*Solyc08g076960*.*1*	1.36	up	K14496	PYL; abscisic acid receptor PYR/PYL family	
**Ethylene**					
*Solyc09g089930*.*1*	3.24	up	K14516	ERF1; ethylene-responsive transcription factor 1	
*Solyc06g053710*.*2*	1.23	up	K14509	ETR, ERS; ethylene receptor	
*Solyc09g075440*.*2*	1.18	up	K14509	ETR, ERS; ethylene receptor	
**Jasmonic acid**					
*Solyc01g005440*.*2*	−1.89	down	K13464	JAZ; jasmonate ZIM domain-containing protein	

### Analysis of oxidative stress genes differentially expressed between hetero- and homografted seedlings

During the normal metabolic processes of plants, photosynthesis and respiration produce reactive oxygen species (ROS)^26^. Under normal growth conditions, the production of ROS in plant cells is maintained at a low level. Environmental stress can cause an increase in the ROS contents. The antioxidant system in plants can remove excess ROS and maintain the normal metabolism of the plant, which involves enzymes such as superoxide dismutase, ascorbate peroxidase, catalase, glutathione reductase, and guaiacol peroxidase, as well as non-enzymatic components, such as ascorbate, glutathione, and other organic acids. In the present study, the significant expression of several candidate genes associated with ROS scavenging, such as glutathione *S*-transferases (*Solyc01g081310*, *Solyc01g099590*, and *Solyc09g007150*), L-ascorbate oxidase (*Solyc12g094460*), and peroxidases (*Solyc02g084800*, *Solyc10g076240*, and *Solyc02g092580*), supported the differential regulation of oxidative stress mechanisms between hetero- and homografted tomato seedlings (Table 6).

**Table 6:** Expression of oxidative stress genes differentially expressed between hetero- and homografted tomato seedlings.

Genes	Log2FoldChange	Up/Down	KEGG:KO	KEGG:Annotation
*Solyc02g084800*.*2*	2.11	up	K00430	peroxidase
*Solyc10g076240*.*1*	2.1	up	—	no
*Solyc01g081310*.*2*	1.91	up	K00799	GST, gst; glutathione S-transferase
*Solyc12g094460*.*1*	1.53	up	—	no
*Solyc08g081690*.*2*	1.33	up	K13447	RBOH; respiratory burst oxidase
*Solyc01g099590*.*2*	1.06	up	K00799	GST, gst; glutathione S-transferase
*Solyc05g053100*.*2*	−1	down	K00382	DLD, lpd, pdhD; dihydrolipoamide dehydrogenase
*Solyc10g082030*.*1*	−1.03	down	K03386	PRDX, ahpC; peroxiredoxin (alkyl hydroperoxide reductase subunit C)
*Solyc02g092580*.*2*	−1.09	down	K00430	peroxidase
*Solyc07g042440*.*2*	−1.1	down	—	no
*Solyc04g074640*.*2*	−1.22	down	—	no
*Solyc09g007150*.*2*	−1.64	down	K00799	GST, gst; glutathione S-transferase

**‘−’** no registration number.

### TFs in the scion of the heterograft combination

**Figure 5:**
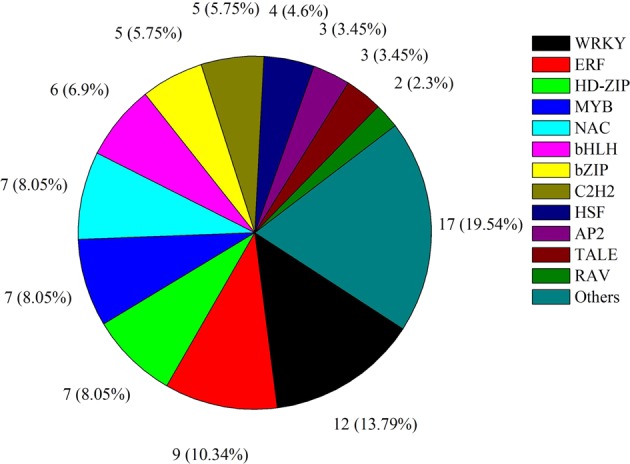
Familial classification of transcription factors differentially expressed in the scion of heterografted tomato seedlings.

TFs are a large group of proteins associated with gene expression and are classified into particular families^27^. TFs are involved in the gene regulation strictly connected with responses to stress; therefore, the genetic manipulation of TFs is highly desirable. In the present analysis, 85 TFs were differentially expressed in the scion of the heterograft combination (Fig. 5). The most differentially expressed TFs were members of the WRKY family (12; 14.12%), followed by ERF (9; 10.59%), NAC (7; 8.24%), MYB (7; 8.27%), HD-ZIP (7; 8.27%), bHLH (6; 7.06%), bZIP (5; 5.88%), and C2H2 (5; 5.88%) families. The majority of the TFs were up-regulated in the scion of the heterograft combination (Supplementary Table S2). This result is consistent with the transcriptome analysis of watermelon grafted onto gourd and pumpkin rootstocks^19^. The WRKY family was initially shown to function in plant defence responses, but more recent findings illustrate that members are involved in the regulation of diverse functional processes, such as growth, development, hormone-mediated pathways, and abiotic stress^28^. WRKY family members are also directly involved in abiotic stress signaling and tolerance. For example, WRKY23 (Solyc01g079260) response to auxin regulation and WRKY70 participates in the defence response to fungus attack. The present results supported the broad functions of this TF gene family in tomato. The functions of other TFs identified are shown in Supplementary Table S2.

### Validation of RNA-Seq data by RT-qPCR analysis

**Figure 6:**
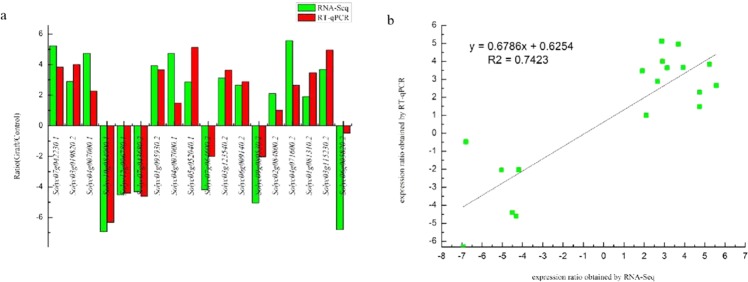
Quantitative real-time PCR (RT-qPCR) validation of selected differentially expressed genes detected between hetero- and homografted tomato seedlings (**a**). Linear regression analysis between gene expression ratios derived from RNA-Seq and RT-qPCR data (**b**). ^**^Significant difference at *P* < 0.05.

To validate the transcript data from the RNA-Seq analysis, we selected 18 DEGs that showed relatively high abundance levels for the RT-qPCR analysis. Total RNAs from the scions of homo- and heterograft combinations were used as the templates. The RT-qPCR data for these genes were generally consistent with the RNA-Seq results (Fig. 6a). A linear regression [(RT-qPCR value) = *a* (RNA-Seq value) + *b*] analysis showed a correlation coefficient of 0.7423, which is indicative of a positive correlation between the RNA-Seq data and the RT-qPCR data (Fig. 6b). Although the observed fold changes differed slightly between the RT-qPCR and RNA-Seq data, this may reflect differences in the sensitivity and specificity of the RT-qPCR and high-throughput sequencing technology. These results indicated that the changes in transcription detected by RNA-Seq were accurate.

## Discussion

Grafting not only is a long-term technique for plant breeding and productivity improvement, but it may also effectively solve the problem of intensive cultivation in a limited cultivatable land area^3,29^. It is also useful for investigating the information exchange between rootstock and scion^12,30^. Early studies of graft morphology showed that the reconnection of vascular bundles between the rootstock and the scion is an important feature of graft healing affinity^16^. In addition, the differentiation of callus cells into vascular tissue to reconnect the xylem and phloem at the graft junction is a vital step for successful grafting^15^. Thus, we observed the anatomical changes during graft healing at five time points (Fig. 1). Our results indicated that the process of cell healing between homo-grafting and hetero-grafting was similar, and healing time had seldom difference between them at 16 DAG. Therefore, there are little differences in the growth and development stages of scions between tomato homo-grafting and hetero-grafting in process of cell healing. Because the grafting healing process of tomato was relatively long, we had tried our best to control the sampling in a relatively relative short time. In addition, we also referred to the experimental method in watermelon grafting study^19^. And, we also tested the physiological and biochemical Indicators (Lignin content, MDA, PPO, etc.) between homo-grafting and hetero-grafting, and found there was no significant difference between them at 16 DAG (data unpublished). Thus, we conducted that the differentially expressed genes between homo-grafting and hetero-grafting scion after 16 DAG were mainly related to the characteristics of scions. therefore, the differences in transcription levels were detected in the scion of them. These differences might be associated with dissimilarities in the translocation of signals and metabolites from the rootstock to the scion^31^.

Based on the enriched GO terms, the overwhelming majority of the DEGs detected in the scion of heterografted tomato were associated with cell parts, response to stimuli, biological regulation, and catalytic activity. This finding illustrated that the transcription of a large number of genes associated with cell rearrangements, cell division, and metabolic patterns was altered in the heterografted tomato seedling. In addition, cells involved in abiotic and biotic stress responses generate an oxidative burst by producing ROS, superoxide anions, hydrogen peroxide, hydroxyl radicals, and nitrous oxide^32^. In the present study, several genes involved in oxidative stress were significantly up-regulated in the scion of heterografted tomato. These data indicated that the rootstock ‘Haomei’ affected the antioxidant defence system in the scion. Furthermore, oxidative stress in graft interfaces has been reported in a number of other plant species, and components of the antioxidant defence system were identified in a transcriptome analysis of hickory (*Carya cathayensis*) after grafting^33^. In tobacco, drought-tolerant rootstock genotypes can improve the drought resistance of the scion by regulating antioxidant enzyme activity and stress-responsive gene expression^33^. For example, the expression of ascorbate peroxidase genes can be switched on by environmental stress. Thus, they are directly engaged in the protection of plants against unfavourable environmental conditions^34^. Alves^35^ used ethylene-insensitive (*Never ripe*; *Nr*) and auxin-insensitive (*dgt*) tomato mutants as rootstocks for the ‘Micro-Tom’ scion and observed that both mutant rootstocks modulated root-to-shoot signalling when responding to antioxidant stress. The present data provided evidence that oxidative stress is involved in the regulatory role of grafting and indicated that antioxidant defence systems were stimulated in the scion of the heterograft combination.

MAPK cascades are ubiquitous signal transduction modules in eukaryotes. These protein phosphorylation cascades link extracellular stimuli to a wide range of cellular responses. In plants, MAPK cascades are involved in responses to diverse biotic and abiotic stresses, phytohormones, cell division, developmental processes^36^, and responses to stresses, such as pathogen infection, wounding, low temperature, drought, osmotic shock, high salinity, and ROS. MAPK pathways also play pivotal roles in processes such as hormonal and developmental signalling. The involvement of the MAPK signalling pathway and phytohormone signal transduction in biotic and abiotic stress responses has often been reported^37–40^. However, relatively few studies have detected the MAPK signalling pathway in response to grafting. A number of metabolic pathways are involved in signalling to heal graft unions^4^. In the current study, a KEGG analysis was carried out to understand the biological functions of the identified DEGs. We observed that the MAPK signalling and plant hormone signal transduction pathways were the most highly enriched pathways among the DEGs, and the majority of their genes were up-regulated in the scion of heterografted tomato. The significant differences in gene expression in the MAPK signalling pathway were associated with pathogen infection, plant hormones, and wounding. Given that the MAPK signalling pathway and phytohormone signal transduction are involved in resistance responses in many crops^41–43^, we hypothesise that these changes might be associated with improvements in the stress resistance in the scion of the heterograft combination.

Plant hormone signal transduction is associated with grafting^44,45^. The present RNA-Seq data showed changes in the expression levels of genes associated with auxin, gibberellin, ABA, ethylene, and jasmonic acid signalling, which play critical roles in the scion of heterografted tomato compared with homografted tomato. Compared with in the homograft combination, the Aux1 family gene *Solyc11g013310*.*1* was inhibited. By modulating the transcript level between the auxin-responsive TFs ARF and AUX, the SAUR protein family gene^46^ *Solyc07g014620*.*1* was significantly up-regulated in heterografted seedlings, which may induce a response in plant growth. The up-regulated expression of the *PIF3* gene *Solyc01g102300*.*2* is associated with gibberellin signalling pathways, which indicated that *PIF3* can be activated by heterografting to regulate the growth and development of tomato. Jasmonates are oxygenated lipids (oxylipins) that regulate, among many other pathways, responses to wounding^47^. Genes associated with jasmonate signalling were down-regulated in the heterografted tomato seedlings compared with homografted tomato seedlings. This result contradicts the findings of Cookson’s^48^ study of heterograft grape. Therefore, the down-regulation of genes involved in jasmonate signalling may also be associated with defence and wound responses during advanced stages of heterograft healing.

## Conclusion

Grafting is particularly important for the cultivation of vegetable crops. An RNA-Seq analysis revealed that 858 genes were significantly differentially expressed in hetero- and homograft tomato combinations, including 528 up-regulated and 330 down-regulated genes. GO and KEGG analyses revealed that the majority of the DEGs in heterografted tomato was associated with primary and secondary metabolism, plant hormone signal transduction, TF regulation, transport, and responses to stimuli. We identified DEGs involved in MAPK signalling, plant hormone signal transduction, and oxidase stress in the scion of heterografted tomato. A number of TFs were up-regulated, such as WRKY, ERF, NAC, and MYB family members. The expression patterns of these genes indicated that the associated biological pathways may be responsible for improved plant performance. This study identified a large number of candidate genes for future functional analyses, and laid a theoretical foundation for an improved understanding of the molecular mechanisms involved in heterografting of tomato to improve scion performance.

## Materials and Methods

### Plant material and microscopic observation of the graft-healing process

Seeds of the tomato cultivars ‘Provence’ and ‘Haomei’ were purchased from Tianjin De Ruiter Seeds Co., Ltd and Shanghai Wells Seed Co., Ltd, respectively. Seedlings were assigned to two groups: scions of ‘Provence’ were grafted onto rootstocks of ‘Haomei’, and homo-grafted ‘Provence’ was used as the control. For the rootstocks, seeds of ‘Haomei’ and ‘Provence’ were sown directly in trays filled with a mixture of peat:vermiculite (3:1, v/v), whereas for the scions ‘Provence’ seeds were sown 7 d later. At the four true-leaves stage, tube grafting was performed^18^. The grafted seedlings were incubated in a phytotron maintained at 28 °C with constant relative humidity of 95% and photosynthetic photon flux density of 50 μmol·m^−2^·s^−1^ for 6 d. The seedlings were then cultivated under a photosynthetic photon flux density of 600 μmol·m^−2^·s^−1^ with a photoperiod of 12 h, temperatures of 25 °C/17 °C (day/night), and a relative humidity of 60%. The developing graft union (the stem segment that includes the grafted portion of the stock and the scion) and the scion (the upper portion of the callus) were sampled at 6, 9, 12, 16, and 21 DAG. The graft-healing process was observed by examining thin sections (8 µm) of paraffin-embedded samples following the method of Kitoh^49^. The sections stained with saffron-solid green dye were observed under an OLYMPUS BX53 Biological Microscope (OLYMPUS BX53). The remaining portion of the scions above the graft union were stored at −80 °C for subsequent RNA extraction and RNA-Seq analysis.

### Processing of RNA-Seq data

Based on observations of the graft-healing process, we sampled the scion from hetero- and homograft combinations (‘Provence’/‘Haomei’ and ‘Provence’/‘Provence’) at 16 DAG for transcriptome sequencing, with three biological replicates per treatment. RNA extraction and RNA-Seq of the samples were performed by the ANOROAD Company (Beijing, China). Raw FASTQ files were deposited in the NCBI Sequence Read Archive under the accession number SUB5937299. Raw sequence reads were processed into clean reads and then filtered to discard low-quality adapter sequences as follows: (1) adapter sequences were removed (the number of bases contaminated by the adaptor in reads was greater than 5 bp; for double-ended sequencing, if one end was contaminated with the adaptor, then the reads at both ends were removed); (2) low-quality reads were discarded (reads with *Q* ≤ 19 accounting for more than 50% of the total bases; for double-ended sequencing, if one end was a low-quality read, then reads at both ends were removed); and (3) reads with a proportion of ambiguous nucleotides (Ns) greater than 5%.

Raw sequence reads were filtered using the Illumina pipeline and were mapped with the RNA-Seq mapping algorithm implemented in HISAT2 v2.1.0 software and reference transcript data for the *S*. *lycopersicum* genome (release ITAG2.4) retrieved from the ENSEMBL website (http://www.ensembl.org/index.html). The procedure allowed alignments with a maximum of two mismatches. The number of mapped clean reads for each gene was counted and then normalised with the DESeq2 v1.20.0 package in R to avoid biases from differences in sequencing depth.

### Identification of differentially expressed genes

To compare gene expression levels between the hetero- and homograft RNA libraries, HTSeq v0.6.0 (http://www-huber.embl.de/users/an ders/HTSeq/doc/overview.html) was used to count the number of reads per gene and to estimate the expression level of each gene. The relative transcript level of each expressed gene was calculated and normalised to the reads per kilobase of transcript per million mapped reads values^50^. The *P*-value threshold was determined by the FDR to account for multiple tests of significance. Screening criteria for the identification of DEGs were as follows: FDR < 0.05 and fold change of reads per kilobase of transcript per million mapped reads > twofold. The DEGs were then subjected to GO functional enrichment and KEGG pathway analyses. The GO terms and KEGG pathways that fulfilled the criterion of a Bonferroni-corrected *P*-value ≤ 0.05 were defined as significantly enriched for the DEGs.

### GO and KEGG analysis

Gene functional annotation was performed based on the tomato genome database (release ITAG2.4) using comparisons of the sequences of the clustered transcriptome assembly with public databases. We performed a BLAST search against the NR and NT (https://www.ncbi.nlm.nih.gov), UniProt (https://www.uniprot.org/), and COG (https://www.ncbi.nlm.nih.gov/COG/) databases with an *E* value cutoff of 10^−5^ to assign putative functions to the DEGs. Sequences with the highest similarities were retrieved for further analysis^51^. KEGG was used to annotate the sequences using metabolic pathways, and Blast2GO was used for the GO classification.

### Quantitative real-time PCR analysis

To verify the accuracy of the RNA-Seq results, 18 DEGs that showed high transcript levels were selected to determine whether their expression levels were consistent with the RNA-Seq results as assessed by RT-qPCR. Total RNA used for RT-qPCR analysis was extracted from the DEGs library in accordance with the manufacturer’s instructions (Nanjing Vazyme BioTech Co., Ltd, Nanjing, China). The RNA extracts were used as templates for RT-qPCR in a LightCycler^®^ 480 real-time PCR system (Roche, Basel, Switzerland). The Power SYBR^®^ Green PCR Master Mix (Applied Biosystems, Foster City, CA, USA) was used as the fluorescence source. Primers were designed with the Primer 3.0 software (http://bioinfo.ut.ee/primer3-0.4.0/). The primer sequences are listed in Supplementary Table S3. The PCR amplification conditions were as follows: 95 °C for 5 min, followed by 45 cycles of 95 °C for 10 s, 60 °C for 30 s, and 72 °C for 30 s. Fluorescent signals were collected at each polymerization step. Three biological replicates and three technical replicates were used per sample. For validation, the RT-qPCR data were calculated as log_2_-fold changes and compared with log_2_-fold values obtained from RNA-Seq^52,53^.

## Supplementary information

Supplementary information

## Data Availability

The authors declare that all the data and plant materials will be available without restrictions.
