# Supplementary material for: De novo Comparative Transcriptome Analysis of Genes Differentially Expressed in the Scion of Homografted and Heterografted Tomato Seedlings
Source: Sci Rep. 2019 Dec 27;9:20240. doi: 10.1038/s41598-019-56563-z (PMC6934607; doi:10.1038/s41598-019-56563-z)
Supplement: Supplementary file 1 — Supplementary information [file 41598_2019_56563_MOESM1_ESM.pdf]

De novo Comparative Transcriptome Analysis of Genes Differentially Expressed in the Scion of Homografted and Heterografted Tomato Seedlings

Peng Zhou[+]·Hui Wang[+]·Wenying Zhu·Wenli Li·FuWang*
College of Horticulture, Qingdao Agricultural University, Qingdao, 266109, China
[+]These authors have contributed equally to this work.
*To whom correspondence should be addressed. E-mail: wangfuabcd@163.com

Supplementary Table S1.KEGG pathway enrichment analysis detected between hetero- and homografted tomato seedlings

Name	Map	Gene_in_DE	Up_Gene	Up_Count	Down_Gene	Down_Count	total gene
Protein processing in endoplasmic reticulum	map04141	gene:Solyc01g0671202|K03347;gene:Solyc01g0795302|K10661;gene:Solyc03g1125902|K13525;gene:Solyc03g1235402|K13993;gene:Solyc04g0144802|K13993;gene:Solyc05g0083902|K10661;gen	gene:Solyc01g067120.2|K03347;gene:Solyc01g079530.2|K10661;gene:Solyc03g112590.2|K13525;gene:Solyc03g123540.2|K13993;gene:Solyc04g014480.2|K13993;gene:Solyc05g008390.2|K10661;	10	.	0	10
Plant hormone signal transduction	map04075	gene:Solyc01g1023002|K12126;gene:Solyc06g0537102|K14509;gene:Solyc07g0146201|K14488;gene:Solyc07g0409902|K14497;gene:Solyc08g0769601|K14496;gene:Solyc08g0777802|K14498;gen	gene:Solyc01g102300.2|K12126;gene:Solyc06g053710.2|K14509;gene:Solyc07g014620.1|K14488;gene:Solyc07g040990.2|K14497;gene:Solyc08g076960.1|K14496;gene:Solyc08g077780.2|K14498;	8	gene:Solyc01g005440.2|K13464;gene:Solyc11g013310.1|K13946	2	10
Photosynthesis - antenna proteins	map00196	gene:Solyc02g0709501|K08912;gene:Solyc02g0709701|K08912;gene:Solyc02g0709801|K08912;gene:Solyc02g0709901|K08912;gene:Solyc02g0710001|K08912;gene:Solyc02g0710101|K08912;gen	.	0	gene:Solyc02g070950.1|K08912;gene:Solyc02g070970.1|K08912;gene:Solyc02g070980.1|K08912;gene:Solyc02g070990.1|K08912;gene:Solyc02g071000.1|K08912;gene:Solyc02g071010.1|K08912;	9	9
MAPK signaling pathway - plant	map04016	gene:Solyc06g0537102|K14509;gene:Solyc07g0409902|K14497;gene:Solyc08g0769601|K14496;gene:Solyc08g0777802|K14498;gene:Solyc08g0816902|K13447;gene:Solyc09g0149902|K13424;gen	gene:Solyc06g053710.2|K14509;gene:Solyc07g040990.2|K14497;gene:Solyc08g076960.1|K14496;gene:Solyc08g077780.2|K14498;gene:Solyc08g081690.2|K13447;gene:Solyc09g014990.2|K13424;	8	.	0	8

Glutathione metabolism	map00480	gene:Solyc01g0813102|K00799;gene:Solyc01g0995902|K00799;gene:Solyc00g18705 02|K01255;gene:Solyc03g0072402|K00797;gene:Solyc05g0057102|K00797;gene:Solyc09g0071502|K00799:gen	gene:Solyc01g081310.2|K00799;gene:Solyc01g099590.2|K00799	2	gene:Solyc00g187050.2|K01255;gene:Solyc03g007240.2|K00797;gene:Solyc05g005710.2|K00797;gene:Solyc09g007150.2|K00799;gene:Solyc12g010020.1|K01255	5	7
Spliceosome	map03040	gene:Solyc01g0998102|K12890;gene:Solyc03g1159202|K12818;gene:Solyc05g0056202|K12822;gene:Solyc07g0227602|K12821;gene:Solyc11g0200401|K03283;gene:Solyc11g0443401|K12821:gen	gene:Solyc01g099810.2|K12890;gene:Solyc03g115920.2|K12818;gene:Solyc05g005620.2|K12822;gene:Solyc07g022760.2|K12821;gene:Solyc11g020040.1|K03283;gene:Solyc11g044340.1|K12821:	7	.	0	7
Biosynthesis of amino acids	map01230	gene:Solyc09g0909002|K01703;gene:Solyc01g0911702|K01476;gene:Solyc03g0834402|K00264;gene:Solyc08g0769702|K01438;gene:Solyc08g0769802|K01438;gene:Solyc11g0076901|K00873:	gene:Solyc09g090900.2|K01703	1	gene:Solyc01g091170.2|K01476;gene:Solyc03g083440.2|K00264;gene:Solyc08g076970.2|K01438;gene:Solyc08g076980.2|K01438;gene:Solyc11g007690.1|K00873	5	6
Carbon metabolism	map01200	gene:Solyc07g0625302|K01595;gene:Solyc01g0940902|K05605;gene:Solyc03g0342202|K01602;gene:Solyc05g0531002|K00382;gene:Solyc07g0436802|K05605;gene:Solyc11g0076901|K00873:	gene:Solyc07g062530.2|K01595	1	gene:Solyc01g094090.2|K05605;gene:Solyc03g034220.2|K01602;gene:Solyc05g053100.2|K00382;gene:Solyc07g043680.2|K05605;gene:Solyc11g007690.1|K00873	5	6

Plant-pathogen interaction	map04626	gene:Solyc04g0181101|K13448;gene:Solyc05g0106702|K09487;gene:Solyc08g0816902|K13447;gene:Solyc09g0149902|K13424;gene:Solyc10g0794201|K13448;gene:Solyc11g0717401|K13448;	gene:Solyc04g018110.1|K13448;gene:Solyc05g010670.2|K09487;gene:Solyc08g081690.2|K13447;gene:Solyc09g014990.2|K13424;gene:Solyc10g079420.1|K13448;gene:Solyc11g071740.1|K13448	6	.	0	6
Ubiquitin mediated proteolysis	map04120	gene:Solyc01g0671202|K03347;gene:Solyc04g0797002|K10260;gene:Solyc08g0679602|K10144;gene:Solyc10g0117402|K06689;gene:Solyc11g0051901|K10143;gene:Solyc10g0805701|K04506;	gene:Solyc01g067120.2|K03347;gene:Solyc04g079700.2|K10260;gene:Solyc08g067960.2|K10144;gene:Solyc10g011740.2|K06689;gene:Solyc11g005190.1|K10143	5	gene:Solyc10g080570.1|K04506	1	6
Monoterpenoid biosynthesis	map00902	gene:Solyc02g0651902|K15099;gene:Solyc02g0903402|K15099;gene:Solyc02g0903502|K15099;gene:Solyc09g0980302|K15099;gene:Solyc11g0714601|K15095;	.	0	gene:Solyc02g065190.2|K15099;gene:Solyc02g090340.2|K15099;gene:Solyc02g090350.2|K15099;gene:Solyc09g098030.2|K15099;gene:Solyc11g071460.1|K15095	5	5
Circadian rhythm - plant	map04712	gene:Solyc01g1023002|K12126;gene:Solyc08g0611302|K16241;gene:Solyc11g0051901|K10143;gene:Solyc12g0566501|K12124;gene:Solyc01g0053002|K12116;	gene:Solyc01g102300.2|K12126;gene:Solyc08g061130.2|K16241;gene:Solyc11g005190.1|K10143;gene:Solyc12g056650.1|K12124	4	gene:Solyc01g005300.2|K12116	1	5

beta-Alanine metabolism	map00410	gene:Solyc06g0649002|K00128;gene:Solyc01g0940902|K05605;gene:Solyc03g0072402|K00797;gene:Solyc05g0057102|K00797;gene:Solyc07g0436802|K05605;	gene:Solyc06g064900.2|K00128	1	gene:Solyc01g094090.2|K05605;gene:Solyc03g007240.2|K00797;gene:Solyc05g005710.2|K00797;gene:Solyc07g043680.2|K05605	4	5
Arginine and proline metabolism	map00330	gene:Solyc06g0649002|K00128;gene:Solyc01g0911702|K01476;gene:Solyc03g0072402|K00797;gene:Solyc05g0057102|K00797;gene:Solyc11g0715501|K01426;	gene:Solyc06g064900.2|K00128	1	gene:Solyc01g091170.2|K01476;gene:Solyc03g007240.2|K00797;gene:Solyc05g005710.2|K00797;gene:Solyc11g071550.1|K01426	4	5
Valine, leucine and isoleucine degradation	map00280	gene:Solyc05g0564802|K00166;gene:Solyc06g0649002|K00128;gene:Solyc01g0940902|K05605;gene:Solyc05g0531002|K00382;gene:Solyc07g0436802|K05605;	gene:Solyc05g056480.2|K00166;gene:Solyc06g064900.2|K00128	2	gene:Solyc01g094090.2|K05605;gene:Solyc05g053100.2|K00382;gene:Solyc07g043680.2|K05605	3	5
Steroid biosynthesis	map00100	gene:Solyc01g0081102|K05917;gene:Solyc01g0913202|K14423;gene:Solyc02g0694902|K09828;gene:Solyc09g0090402|K00222;	.	0	gene:Solyc01g008110.2|K05917;gene:Solyc01g091320.2|K14423;gene:Solyc02g069490.2|K09828;gene:Solyc09g009040.2|K00222	4	4

Propanoate metabolism	map00640	gene:Solyc05g0564802|K00166;gene:Solyc01g0940902|K05605;gene:Solyc05g0531002|K00382;gene:Solyc07g0436802|K05605;	gene:Solyc05g056480.2|K00166	1	gene:Solyc01g094090.2|K05605;gene:Solyc05g053100.2|K00382;gene:Solyc07g043680.2|K05605	3	4
Pyruvate metabolism	map00620	gene:Solyc06g0649002|K00128;gene:Solyc07g0625302|K01595;gene:Solyc05g0531002|K00382;gene:Solyc11g0076901|K00873;	gene:Solyc06g064900.2|K00128;gene:Solyc07g062530.2|K01595	2	gene:Solyc05g053100.2|K00382;gene:Solyc11g007690.1|K00873	2	4
Ribosome	map03010	gene:Solyc01g1035102|K02925;gene:Solyc03g0979502|K02904;gene:Solyc12g0988901|K02881;gene:Solyc12g1001601|K02933;	.	0	gene:Solyc01g103510.2|K02925;gene:Solyc03g097950.2|K02904;gene:Solyc12g098890.1|K02881;gene:Solyc12g100160.1|K02933	4	4
Zeatin biosynthesis	map00908	gene:Solyc01g0801502|K10760;gene:Solyc05g0534001|K13492;gene:Solyc10g0793501|K13492;gene:Solyc11g0666701|K13492;	gene:Solyc01g080150.2|K10760	1	gene:Solyc05g053400.1|K13492;gene:Solyc10g079350.1|K13492;gene:Solyc11g066670.1|K13492	3	4

Arginine biosynthesis	map00220	gene:Solyc01g0911702|K01476;gene:Solyc08g0769702|K01438;gene:Solyc08g0769802|K01438;	.	0	gene:Solyc01g091170.2|K01476;gene:Solyc08g076970.2|K01438;gene:Solyc08g076980.2|K01438	3	3
Carotenoid biosynthesis	map00906	gene:Solyc01g1082102|K09843;gene:Solyc08g0056102|K09843;gene:Solyc06g0846102|K17911;	gene:Solyc01g108210.2|K09843;gene:Solyc08g005610.2|K09843	2	gene:Solyc06g084610.2|K17911	1	3
Porphyrin and chlorophyll metabolism	map00860	gene:Solyc04g0768702|K02492;gene:Solyc06g0603102|K13600;gene:Solyc11g0128501|K13600;	.	0	gene:Solyc04g076870.2|K02492;gene:Solyc06g060310.2|K13600;gene:Solyc11g012850.1|K13600	3	3
Galactose metabolism	map00052	gene:Solyc01g0791702|K18819;gene:Solyc02g0865302|K06617;gene:Solyc07g0659802|K06617;	gene:Solyc01g079170.2|K18819;gene:Solyc02g086530.2|K06617;gene:Solyc07g065980.2|K06617	3	.	0	3

Terpenoid backbone biosynthesis	map00900	gene:Solyc00g1365602|K00806;gene:Solyc07g0619902|K05356;gene:Solyc10g0851401|K11778;	gene:Solyc00g136560.2|K00806;gene:Solyc07g061990.2|K05356;gene:Solyc10g085140.1|K11778	3	.	0	3
2-Oxocarboxylic acid metabolism	map01210	gene:Solyc09g0909002|K01703;gene:Solyc08g0769702|K01438;gene:Solyc08g0769802|K01438;	gene:Solyc09g090900.2|K01703	1	gene:Solyc08g076970.2|K01438;gene:Solyc08g076980.2|K01438	2	3
Amino sugar and nucleotide sugar metabolism	map00520	gene:Solyc12g0102001|K13648;gene:Solyc03g1153801|K00012;gene:Solyc07g0050902|K01183;	gene:Solyc12g010200.1|K13648	1	gene:Solyc03g115380.1|K00012;gene:Solyc07g005090.2|K01183	2	3
Cutin, suberine and wax biosynthesis	map00073	gene:Solyc01g0959302|K15406;gene:Solyc03g0652502|K15404;gene:Solyc07g0538902|K15406;	gene:Solyc01g095930.2|K15406;gene:Solyc03g065250.2|K15404;gene:Solyc07g053890.2|K15406	3	.	0	3

Cysteine and methionine metabolism	map00270	gene:Solyc07g0495302|K05933;gene:Solyc03g0072402|K00797;gene:Solyc05g0057102|K00797;	gene:Solyc07g049530.2|K05933	1	gene:Solyc03g007240.2|K00797;gene:Solyc05g005710.2|K00797	2	3
Glycolysis / Gluconeogenesis	map00010	gene:Solyc06g0649002|K00128;gene:Solyc05g0531002|K00382;gene:Solyc11g0076901|K00873;	gene:Solyc06g064900.2|K00128	1	gene:Solyc05g053100.2|K00382;gene:Solyc11g007690.1|K00873	2	3
Glyoxylate and dicarboxylate metabolism	map00630	gene:Solyc03g0342202|K01602;gene:Solyc05g0531002|K00382;gene:Solyc05g0558102|K01091;	.	0	gene:Solyc03g034220.2|K01602;gene:Solyc05g053100.2|K00382;gene:Solyc05g055810.2|K01091	3	3
Phenylpropanoid biosynthesis	map00940	gene:Solyc01g0103902|K01188;gene:Solyc02g0848002|K00430;gene:Solyc02g0925802|K00430;	gene:Solyc01g010390.2|K01188;gene:Solyc02g084800.2|K00430	2	gene:Solyc02g092580.2|K00430	1	3

Nitrogen metabolism	map00910	gene:Solyc02g0677902|K02575;gene:Solyc03g0834402|K00264;	gene:Solyc02g067790.2|K02575	1	gene:Solyc03g083440.2|K00264	1	2
ABC transporters	map02010	gene:Solyc02g0874102|K05658;gene:Solyc03g0058602|K05658;	gene:Solyc02g087410.2|K05658	1	gene:Solyc03g005860.2|K05658	1	2
Alanine, aspartate and glutamate metabolism	map00250	gene:Solyc07g0433102|K16871;gene:Solyc03g0834402|K00264;	gene:Solyc07g043310.2|K16871	1	gene:Solyc03g083440.2|K00264	1	2
alpha-Linolenic acid metabolism	map00592	gene:Solyc01g0065602|K00454;gene:Solyc11g0698001|K01723;	gene:Solyc01g006560.2|K00454	1	gene:Solyc11g069800.1|K01723	1	2

Ascorbate and aldarate metabolism	map00053	gene:Solyc06g0649002|K00128;gene:Solyc03g1153801|K00012;	gene:Solyc06g064900.2|K00128	1	gene:Solyc03g115380.1|K00012	1	2
Carbon fixation in photosynthetic organisms	map00710	gene:Solyc07g0625302|K01595;gene:Solyc03g0342202|K01602;	gene:Solyc07g062530.2|K01595	1	gene:Solyc03g034220.2|K01602	1	2
Diterpenoid biosynthesis	map00904	gene:Solyc02g0801201|K04125;gene:Solyc10g0053602|K04125;	gene:Solyc02g080120.1|K04125;gene:Solyc10g005360.2|K04125	2	.	0	2
Fatty acid elongation	map00062	gene:Solyc10g0092402|K15397;gene:Solyc12g0967801|K07512;	gene:Solyc10g009240.2|K15397	1	gene:Solyc12g096780.1|K07512	1	2

Fatty acid metabolism	map01212	gene:Solyc12g0092601|K09458;gene:Solyc12g0967801|K07512;	.	0	gene:Solyc12g009260.1|K09458;gene:Solyc12g096780.1|K07512	2	2
Glycerolipid metabolism	map00561	gene:Solyc06g0649002|K00128;gene:Solyc10g0849001|K13508;	gene:Solyc06g064900.2|K00128	1	gene:Solyc10g084900.1|K13508	1	2
Glycerophospholipid metabolism	map00564	gene:Solyc02g0684302|K00968;gene:Solyc10g0849001|K13508;	gene:Solyc02g068430.2|K00968	1	gene:Solyc10g084900.1|K13508	1	2
Isoquinoline alkaloid biosynthesis	map00950	gene:Solyc02g0786502|K00422;gene:Solyc07g0083801|K13393;	gene:Solyc02g078650.2|K00422	1	gene:Solyc07g008380.1|K13393	1	2

Linoleic acid metabolism	map00591	gene:Solyc01g0065602|K00454;gene:Solyc08g0140002|K15718;	gene:Solyc01g006560.2|K00454	1	gene:Solyc08g014000.2|K15718	1	2
Pentose and glucuronate interconversions	map00040	gene:Solyc01g0910502|K01051;gene:Solyc03g1153801|K00012;	gene:Solyc01g091050.2|K01051	1	gene:Solyc03g115380.1|K00012	1	2
Peroxisome	map04146	gene:Solyc05g0543202|K08726;gene:Solyc11g0129901|K13348;	.	0	gene:Solyc05g054320.2|K08726;gene:Solyc11g012990.1|K13348	2	2
Phagosome	map04145	gene:Solyc04g0076502|K00921;gene:Solyc05g0562302|K08057;	gene:Solyc04g007650.2|K00921;gene:Solyc05g056230.2|K08057	2	.	0	2

Photosynthesis	map00195	gene:Solyc06g0829502|K02699;gene:Solyc10g0751601|K02639;	.	0	gene:Solyc06g082950.2|K02699;gene:Solyc10g075160.1|K02639	2	2
Purine metabolism	map00230	gene:Solyc02g0784002|K01466;gene:Solyc11g0076901|K00873;	gene:Solyc02g078400.2|K01466	1	gene:Solyc11g007690.1|K00873	1	2
Ribosome biogenesis in eukaryotes	map03008	gene:Solyc01g1099902|K14573;gene:Solyc02g0896002|K14545;	gene:Solyc01g109990.2|K14573;gene:Solyc02g089600.2|K14545	2	.	0	2
Starch and sucrose metabolism	map00500	gene:Solyc01g0103902|K01188;gene:Solyc07g0065002|K16055;	gene:Solyc01g010390.2|K01188;gene:Solyc07g006500.2|K16055	2	.	0	2

Tropane, piperidine and pyridine alkaloid biosynthesis	map00960	gene:Solyc06g0834702|K08081;gene:Solyc06g0839102|K12692;	.	0	gene:Solyc06g083470.2|K08081;gene:Solyc06g083910.2|K12692	2	2
Tryptophan metabolism	map00380	gene:Solyc06g0649002|K00128;gene:Solyc11g0715501|K01426;	gene:Solyc06g064900.2|K00128	1	gene:Solyc11g071550.1|K01426	1	2
Tyrosine metabolism	map00350	gene:Solyc02g0786502|K00422;gene:Solyc12g0141001|K00451;	gene:Solyc02g078650.2|K00422;gene:Solyc12g014100.1|K00451	2	.	0	2
Arachidonic acid metabolism	map00590	gene:Solyc05g0543202|K08726;	.	0	gene:Solyc05g054320.2|K08726	1	1

Basal transcription factors	map03022	gene:Solyc07g0068202|K03125;	gene:Solyc07g006820.2|K03125	1	.	0	1
Base excision repair	map03410	gene:Solyc10g0052102|K01246;	gene:Solyc10g005210.2|K01246	1	.	0	1
Biotin metabolism	map00780	gene:Solyc12g0092601|K09458;	.	0	gene:Solyc12g009260.1|K09458	1	1
Brassinosteroid biosynthesis	map00905	gene:Solyc10g0865001|K09591;	.	0	gene:Solyc10g086500.1|K09591	1	1

Butanoate metabolism	map00650	gene:Solyc07g0433102|K16871;	gene:Solyc07g043310.2|K16871	1	.	0	1
C5-Branched dibasic acid metabolism	map00660	gene:Solyc09g0909002|K01703;	gene:Solyc09g090900.2|K01703	1	.	0	1
Cholesterol metabolism	map04979	gene:Solyc03g1132102|K15040;	.	0	gene:Solyc03g113210.2|K15040	1	1
Citrate cycle (TCA cycle)	map00020	gene:Solyc05g0531002|K00382;	.	0	gene:Solyc05g053100.2|K00382	1	1

Cyanoamino acid metabolism	map00460	gene:Solyc01g0103902|K01188;	gene:Solyc01g010390.2|K01188	1	.	0	1
DNA replication	map03030	gene:Solyc07g0183002|K10739;	.	0	gene:Solyc07g018300.2|K10739	1	1
Endocytosis	map04144	gene:Solyc11g0200401|K03283;	gene:Solyc11g020040.1|K03283	1	.	0	1
Fatty acid biosynthesis	map00061	gene:Solyc12g0092601|K09458;	.	0	gene:Solyc12g009260.1|K09458	1	1

Fatty acid degradation	map00071	gene:Solyc06g0649002|K00128;	gene:Solyc06g064900.2|K00128	1	.	0	1
Flavonoid biosynthesis	map00941	gene:Solyc05g0098601|K13081;	.	0	gene:Solyc05g009860.1|K13081	1	1
Glucosinolate biosynthesis	map00966	gene:Solyc09g0909002|K01703;	gene:Solyc09g090900.2|K01703	1	.	0	1
Glycine, serine and threonine metabolism	map00260	gene:Solyc05g0531002|K00382;	.	0	gene:Solyc05g053100.2|K00382	1	1

Histidine metabolism	map00340	gene:Solyc06g0649002|K00128;	gene:Solyc06g064900.2|K00128	1	.	0	1
Homologous recombination	map03440	gene:Solyc07g0183002|K10739;	.	0	gene:Solyc07g018300.2|K10739	1	1
Inositol phosphate metabolism	map00562	gene:Solyc04g0076502|K00921;	gene:Solyc04g007650.2|K00921	1	.	0	1
Isoflavonoid biosynthesis	map00943	gene:Solyc04g0052302|K13258;	.	0	gene:Solyc04g005230.2|K13258	1	1

Lysine degradation	map00310	gene:Solyc06g0649002|K00128;	gene:Solyc06g064900.2|K00128	1	.	0	1
Mismatch repair	map03430	gene:Solyc07g0183002|K10739;	.	0	gene:Solyc07g018300.2|K10739	1	1
Nucleotide excision repair	map03420	gene:Solyc07g0183002|K10739;	.	0	gene:Solyc07g018300.2|K10739	1	1
Phenylalanine metabolism	map00360	gene:Solyc11g0715501|K01426;	.	0	gene:Solyc11g071550.1|K01426	1	1

Phosphatidylinositol signaling system	map04070	gene:Solyc04g0076502|K00921;	gene:Solyc04g007650.2|K00921	1	.	0	1
Phosphonate and phosphinate metabolism	map00440	gene:Solyc02g0684302|K00968;	gene:Solyc02g068430.2|K00968	1	.	0	1
Riboflavin metabolism	map00740	gene:Solyc03g0980102|K14379;	.	0	gene:Solyc03g098010.2|K14379	1	1
Sesquiterpenoid and triterpenoid biosynthesis	map00909	gene:Solyc12g0065301|K15813;	gene:Solyc12g006530.1|K15813	1	.	0	1

Ubiquinone and other terpenoid-quinone biosynthesis	map00130	gene:Solyc07g0177702|K09833;	.	0	gene:Solyc07g017770.2|K09833	1	1
Valine, leucine and isoleucine biosynthesis	map00290	gene:Solyc09g0909002|K01703;	gene:Solyc09g090900.2|K01703	1	.	0	1
Vitamin B6 metabolism	map00750	gene:Solyc03g1214202|K13248;	.	0	gene:Solyc03g121420.2|K13248	1	1

Supplementary Table S2. Familial classification of transcription factors differentially expressed in the scion of heterografted tomato seedlings

Gene		Reference_Spe	Family	Ref_Gene_Introduction
Solyc06g0755	down	Solanum_lyco	AP2	related to AP2.7
Solyc09g0072	down	Solanum_lyco	AP2	related to AP2.7
Solyc10g0843	down	Solanum_lyco	AP2	related to AP2.7
Solyc01g1023	up	Solanum_lyco	bHLH	phytochrome interacting factor 3
Solyc03g0978	up	Solanum_lyco	bHLH	basic helix-loop-helix (bHLH) DNA-binding superfamily protein
Solyc03g1183	down	Solanum_lyco	bHLH	basic helix-loop-helix (bHLH) DNA-binding superfamily protein
Solyc03g1193	up	Solanum_lyco	bHLH	BR enhanced expression 3
Solyc03g1212	up	Solanum_lyco	bHLH	basic helix-loop-helix (bHLH) DNA-binding superfamily protein
Solyc09g0577	up	Solanum_lyco	bHLH	basic helix-loop-helix (bHLH) DNA-binding superfamily protein
Solyc02g0619	up	Solanum_lyco	bZIP	basic region/leucine zipper motif 27
Solyc02g0629	up	Solanum_lyco	bZIP	G-box binding factor 1
Solyc02g0920	up	Solanum_lyco	bZIP	basic leucine-zipper 6
Solyc08g0611	up	Solanum_lyco	bZIP	Basic-leucine zipper (bZIP) transcription factor family protein
Solyc10g0540	up	Solanum_lyco	bZIP	basic leucine-zipper 44
Solyc04g0085	up	Solanum_lyco	C2H2	indeterminate(ID)-domain 4
Solyc04g0779	up	Solanum_lyco	C2H2	salt tolerance zinc finger
Solyc05g0091	up	Solanum_lyco	C2H2	C2H2 and C2HC zinc fingers superfamily protein
Solyc08g0630	up	Solanum_lyco	C2H2	indeterminate(ID)-domain 11
Solyc09g0075	up	Solanum_lyco	C2H2	C2H2-like zinc finger protein
Solyc06g0630	up	Solanum_lyco	C3H	SAP domain-containing protein
Solyc07g0451	up	Solanum_lyco	CO-like	B-box type zinc finger protein with CCT domain
Solyc02g0786	up	Solanum_lyco	Dof	Dof-type zinc finger DNA-binding family protein
Solyc03g0935	up	Solanum_lyco	ERF	Integrase-type DNA-binding superfamily protein
Solyc03g0935	up	Solanum_lyco	ERF	Integrase-type DNA-binding superfamily protein
Solyc03g0935	up	Solanum_lyco	ERF	Integrase-type DNA-binding superfamily protein
Solyc04g0729	up	Solanum_lyco	ERF	related to AP2 4
Solyc05g0520	up	Solanum_lyco	ERF	Integrase-type DNA-binding superfamily protein
Solyc05g0520	up	Solanum_lyco	ERF	Integrase-type DNA-binding superfamily protein
Solyc05g0520	up	Solanum_lyco	ERF	ethylene responsive element binding factor 1
Solyc08g0822	up	Solanum_lyco	ERF	Integrase-type DNA-binding superfamily protein
Solyc09g0899	up	Solanum_lyco	ERF	ethylene response factor 1

Solyc08g0764(	up	Solanum_lycoj	G2-like	Homeodomain-like superfamily protein
Solyc12g0088:	up	Solanum_lycoj	GATA	cytokinin-responsive gata factor 1
Solyc06g0831!	up	Solanum_lycoj	GeBP	FKBP-like peptidyl-prolyl cis-trans isomerase family protein
Solyc01g1002(	up	Solanum_lycoj	GRAS	SCARECROW-like 14
Solyc01g0739	up	Solanum_lycoj	HD-ZIP	homeobox from Arabidopsis thaliana
Solyc02g0802(	down	Solanum_lycoj	HD-ZIP	protodermal factor 2
Solyc02g0869:	up	Solanum_lycoj	HD-ZIP	homeobox 1
Solyc02g0919:	up	Solanum_lycoj	HD-ZIP	Homeobox-leucine zipper protein family
Solyc04g0772:	up	Solanum_lycoj	HD-ZIP	Homeobox-leucine zipper protein family
Solyc06g0359	down	Solanum_lycoj	HD-ZIP	Homeobox-leucine zipper family protein / lipid-binding START domain-containing protein
Solyc08g0783(	up	Solanum_lycoj	HD-ZIP	homeobox protein 2
Solyc02g0908:	up	Solanum_lycoj	HSF	heat shock factor 4
Solyc06g0539(	up	Solanum_lycoj	HSF	heat shock transcription factor A6B
Solyc08g0629(	up	Solanum_lycoj	HSF	heat shock transcription factor A2
Solyc09g0656(	up	Solanum_lycoj	HSF	heat shock transcription factor A6B
Solyc03g1195:	up	Solanum_lycoj	LBD	LOB domain-containing protein 41
Solyc01g1023	up	Solanum_lycoj	MYB	myb domain protein 61
Solyc06g0716!	up	Solanum_lycoj	MYB	myb domain protein 86
Solyc06g0839(	down	Solanum_lycoj	MYB	myb domain protein 13
Solyc12g0493:	up	Solanum_lycoj	MYB	myb domain protein 12
Solyc01g0739!	up	Solanum_lycoj	MYB_relat	DNA-binding bromodomain-containing protein
Solyc03g1136:	up	Solanum_lycoj	MYB_relat	Homeodomain-like superfamily protein
Solyc06g0756(	up	Solanum_lycoj	MYB_relat	myb domain protein 48
Solyc01g0098(	up	Solanum_lycoj	NAC	NAC domain containing protein 83
Solyc02g0934:	up	Solanum_lycoj	NAC	NAC domain containing protein 44
Solyc04g0056	up	Solanum_lycoj	NAC	NAC-like, activated by AP3/PI
Solyc04g0799	up	Solanum_lycoj	NAC	NAC domain containing protein 38
Solyc05g0077	up	Solanum_lycoj	NAC	NAC-like, activated by AP3/PI
Solyc08g0086(	up	Solanum_lycoj	NAC	NAC (No Apical Meristem) domain transcriptional regulator superfamily protein
Solyc10g0557(	up	Solanum_lycoj	NAC	NAC domain containing protein 36
Solyc04g0070(	up	Solanum_lycoj	RAV	related to ABI3/VP1 1
Solyc05g0097!	up	Solanum_lycoj	RAV	AP2/B3 transcription factor family protein
Solyc02g0779:	up	Solanum_lycoj	SBP	squamosa promoter binding protein-like 3
Solyc01g0070	up	Solanum_lycoj	TALE	BEL1-like homeodomain 1
Solyc04g0772	up	Solanum_lycoj	TALE	KNOTTED-like from Arabidopsis thaliana
Solyc11g0698!	up	Solanum_lycoj	TALE	BEL1-like homeodomain 8
Solyc03g0068(	down	Solanum_lycoj	TCP	TCP family transcription factor
Solyc12g0141	down	Solanum_lycoj	TCP	TCP family transcription factor 4
Solyc12g0565	up	Solanum_lycoj	Trihelix	Duplicated homeodomain-like superfamily protein
Solyc01g0792(	up	Solanum_lycoj	WRKY	WRKY DNA-binding protein 23
Solyc01g0956:	up	Solanum_lycoj	WRKY	WRKY family transcription factor
Solyc02g0216!	up	Solanum_lycoj	WRKY	WRKY DNA-binding protein 14

Solyc02g0721	up	Solanum_lyco	WRKY	WRKY DNA-binding protein 65
Solyc03g0957	up	Solanum_lyco	WRKY	WRKY DNA-binding protein 70
Solyc03g1168	up	Solanum_lyco	WRKY	WRKY DNA-binding protein 40
Solyc04g0785	up	Solanum_lyco	WRKY	WRKY DNA-binding protein 7
Solyc05g0557	up	Solanum_lyco	WRKY	WRKY DNA-binding protein 26
Solyc06g0684	up	Solanum_lyco	WRKY	WRKY DNA-binding protein 40
Solyc08g0673	up	Solanum_lyco	WRKY	WRKY DNA-binding protein 40
Solyc09g0149	up	Solanum_lyco	WRKY	WRKY DNA-binding protein 33
Solyc09g0157	up	Solanum_lyco	WRKY	WRKY DNA-binding protein 70
Solyc01g0910	down	Solanum_lyco	YABBY	Plant-specific transcription factor YABBY family protein
Solyc02g0851	up	Solanum_lyco	ZF-HD	homeobox protein 24
Solyc03g1160	up	Solanum_lyco	ZF-HD	mini zinc finger

Supplementary table S3.Quantitative real-time PCR (RT-qPCR) validated primer sequences for selected differentially expressed genes detected between hetero- and homografted tomato seedlings

No.	Gene	Log2FoldChange	Up/Down	Forward primer (5' -3')	Reverse primer (5' -3')
1	*Solyc07g042230.1*	5.23	up	GCGGCTAGAGCTTATGATGC	TGTCTGGATGCCTTCACTTG
2	*Solyc03g019820.2*	2.90	up	ATCCAGATACGGGGTTAGGG	CCCACAAGTGAACCAAAGGT
3	*Solyc04g007000.1*	4.74	up	TATGATATCGCGGCACAGAG	TCTTGGCATGTTGAAGTTCG
4	*Solyc10g084900.1*	-6.94	down	GCCACCCCTAGGATAATGGT	GAACTCCGGGTGGTCTAACA
5	*Solyc12g096780.1*	-4.52	down	TCATCTATTGGCCCTCGTTC	TGTTTTCCCATTGCCTTTTC
6	*Solyc07g043680.2*	-4.32	down	GAGAAGGAAGGACCCAAACC	CAGCCCTCATTCCCTCATAA
7	*Solyc01g095930.2*	3.93	up	TTGAAGGATTCCCAATCACC	AGTTGCGTTTGTGGCATTTT
8	*Solyc04g007000.1*	4.74	up	TTCCAAAACAACACGCAGAG	ATCGAAATCGCCACATTTTC
9	*Solyc05g052040.1*	2.87	up	GCAGCATTTAGGCTCAGAGG	TCTCACCCTTTTCCCACAAG
10	*Solyc07g064600.2*	-4.19	down	CACCGGGAAGCTTTTGTATG	CCGGTACTGGCTAAGCAAAC
11	*Solyc03g123540.2*	3.13	up	CCGTGGACATTCTGGATACC	CCAGCGTCTTCTCATCTTCC
12	*Solyc06g009140.2*	2.66	up	TATCCAGAGGCGTGGATACG	TTCAGCAGCATCAATTTCCTT
13	*Solyc09g089530.2*	-5.04	down	GTGGCCAGAACTTATTGGTG	TAACCAACCACAGGCATTGA
14	*Solyc02g084800.2*	2.11	up	TTTGTTGAGGCTCCATTTCC	TTTGTGGGCATTCTTTCTCC
15	*Solyc04g071600.2*	5.57	up	AGCACCATAGCCATCTCCAG	ATGGAATGCAAATCCACCAG
16	*Solyc01g081310.2*	1.91	up	CCGGTTTTGGTTCATAATGG	CCTGGCACACACTTGTCATC
17	*Solyc03g115230.2*	3.69	up	ACCTCTGCATATGGCTTTGG	CAGGTGCTGGTGACTGAGAA
18	*Solyc06g005820.2*	-6.80	down	GAGGAAGCGGAATGATGATG	CGCCATGAAGAAGACAACAA
19	*actin*			CAAACGAGAATTGCCTTGGT	CTTAACATCCGCACCAACCT
